# Impact of a virtual reality-based intervention on motor performance and balance of a child with cerebral palsy: a case study

**DOI:** 10.1016/j.rpped.2014.04.005

**Published:** 2014-12

**Authors:** Silvia Leticia Pavão, Joice Luiza Bruno Arnoni, Alyne Kalyane Câmara de Oliveira, Nelci Adriana Cicuto Ferreira Rocha

**Affiliations:** Universidade Federal de São Carlos (UFSCar), São Carlos, SP, Brazil

**Keywords:** Cerebral palsy, Children, Postural balance, Psychomotor performance, Rehabilitation, Virtual reality exposure therapy

## Abstract

**OBJECTIVE:**

To verify the effect of an intervention protocol using virtual reality (VR) on the motor performance and balance of a child with cerebral palsy (CP).

**CASE DESCRIPTION:**

To comply with the proposed objectives, a 7-year old child with spastic hemiplegic cerebral palsy (CP), GMFCS level I, was submitted to a physiotherapy intervention protocol of 12 45-minute sessions, twice a week, using virtual reality-based therapy. The protocol used a commercially-available console (*XBOX(r)360 *Kinect^(r))^ able to track and reproduce body movements on a screen. Prior to the intervention protocol, the child was evaluated using the Motor Development Scale (MDS) and the Pediatric Balance Scale (PBS) in order to assess motor development and balance, respectively. Two baseline assessments with a 2-week interval between each other were carried out for each tool. Then, the child was re-evaluated after the twelfth session. The results showed no changes in the two baseline scores. After the intervention protocol, the child improved his scores in both tools used: the PBS score increased by 3 points, reaching the maximal score, and the MDS increased from a much inferior motor performance to just an inferior motor performance.

**COMMENTS:**

The evidence presented in this case supports the use of virtual reality as a promising tool to be incorporated into the rehabilitation process of patients with neuromotor dysfunction.

## Introduction

Acquired brain injuries, such as hypoxic-ischemic lesions up to the age of three, are among the ten main causes of spastic hemiplegic cerebral palsy (CP).^1^^-^ 2 Although it does not severely impair functionality in children, hemiplegic motor impairment produces neuromotor alterations that cause precision deficits in movement performance and deficits in postural control, which is responsible for the stability and alignment between the body segments during the performance of activities.^3^ Thus, the rehabilitation of children with mild motor impairment of the hemiplegic type may prove to be especially challenging to therapists, requiring profound technical knowledge and creativity.^4^^-^ 5

The presence of neuromotor impairments in hemiplegic CP and its high incidence in the pediatric age range^5^ justify the development of studies assessing the effect of therapeutic interventions on the balance and quality of postural responses in these children,^6^^-^ 8 aiming to determine the most effective approaches in functional recovery. In this sense, virtual reality (VR)-based therapy is an increasingly acknowledged interactive tool used for patient immersion in a virtual environment.^9^^-^ 11

VR provides patients with simplified feedback about the position of their bodies in space^12^ and allows them to interact with virtual components in real time,^13^ stimulating the learning of adaptive motor control strategies in response to stimuli.^8^^,^ 14 The motivation and the ability to customize the therapy provided by the contact with the virtual environment^15^^,^ 16 make VR an important rehabilitation tool, which offers sensorimotor experiences that are otherwise unfeasible in common therapies. 

Studies have shown improvement in manual function and cortical organization in children with CP after VR-based therapies;^17^^-^ 18 however, although there are studies using VR-based therapy in children with CP, most of them use interfaces with the virtual environment through gloves^17^ or controls with accelerometers.^18^^-^ 20 Few studies have used VR in the rehabilitation of children with CP through the use of commercial body movement tracking systems.^21^^-^ 23

Commercial body movement tracking systems such as the XBOX^(r)^360 Kinect(r) (Microsoft(r), New York, United States) contain sensors that capture the child's movements in three dimensions, creating images that can be viewed by the individual on a TV screen. This projection allows interaction with the virtual environment through body movement. This is an interesting feature, considering the upper limb impairment seen in children with CP, which could prevent the use of gloves and remote controls in their treatment. Chang *et al* 22 observed the effect of VR-based therapy using the XBOX^(r)^360 Kinect(r) on hand function of children with CP. The authors used specific movements for the upper limbs, checking positive effects of this treatment modality in children with moderate motor impairment. Luna-Oliva *et al* 23 also found positive effects of using the Kinect as an adjunct therapeutic tool on motor function in children with mild and moderate motor impairment. 

However, although these studies have demonstrated the benefits of using video games with body motion tracking as a therapeutic adjuvant,^22^^-^ 23 the evidence found is still limited, especially regarding its use in children with low functional impairment. According to Campos *et al*,^4^ the lower the motor impairment observed in children with CP, the more difficult it is to obtain therapeutic gains in the rehabilitation process. Therefore, the progression of the therapy in these children is often compromised by the difficulty in finding tasks that motivate them, while at the same time showing therapeutic efficacy. Considering all these factors, we report a case that evaluated the effect of an intervention using VR through the commercially available XBOX^(r)^360 Kinect(r) console (Microsoft(r), New York, United States) on the motor performance and functional balance of a child with mild motor impairment CP. Although this is a preliminary assessment, it is believed that the immersion afforded by the contact with the virtual environment, together with motivation arising from the contact with the game, allow the child with mild motor impairment and high levels of functionality to perform tasks close to those performed in their daily routine, facilitating the transposition of the motor learning generated during therapy and leading to measurable functional gains, increasing the social integration to the environment that surrounds them.^14^^,^ 24

## Case report

M.N.S., seven years old, male, has right spastic hemiplegic CP, level I, according to the Gross Motor Function Classification System (GMFCS),^25^ caused by a hypoxic-ischemic injury at two years old. The child has participated in a physical therapy intervention program based on the Bobath Neuroevolutive Concept twice a week for four years. His main complaint is the difficulty in participating in games that include jumping and overcoming obstacles at school with his friends. 

The proposed intervention was approved by the Ethics Committee on Human Research of the institution where the research took place (Case N. 190 903), in accordance with Resolution 196/96 of the National Health Council. The child's guardian authorized his participation in the study by signing the Free and Informed Consent form.

The study involved the use of multiple repeated measures in a single subject, according to the A-B-A model.^21^^-^ 23 A trained physical therapist, blinded to the study, performed all assessments during the three phases of the study: baseline 1 (B1), two weeks prior to the trial; baseline 2 (B2), on the day immediately prior to the intervention period; baseline 3 (B3), on the day after the intervention was finished. Assessments of the child's motor performance through the Motor Development Scale (MDS),^26^ and of the functional balance through the Pediatric Balance Scale (PBS) were performed^27^. 

The MDS tool assesses the following areas of development: fine motor skills (MA1), gross motor skills (MA2), balance (MA3), body schema (MA4), spatial organization (MA5), temporal organization (MA6) and laterality. With the exception of laterality, the other areas are evaluated by ten tasks each, distributed in progressive difficulty, between the ages of 2 and 11 years. The child should start with the tasks related to the age immediately below theirs. Each successfully completed task determines one point in the correspondent Motor Age (MA). Based on the calculation of the motor ages in each area, the calculation of the General Motor Quotient (GMQ) is carried out. The values ​​of GMQ are quantified and categorized, allowing the classification of analyzed skills as: much superior (130 or more), superior (120-129), high-normal (110-119), medium-normal (90-109), low-normal (80-89), inferior (70-79), and much inferior (69 or less). 

The PBS questionnaire, adapted from the Berg Balance Scale, assesses the balance in children with neuromotor dysfunction.^27^ It consists of 14 activities, scored from zero to four, with zero indicating maximum or full assistance to perform the activity, and four indicating the independent performance of a task. The total score is the sum of all the scores, ranging from zero to 56, with higher scores indicating better skills in balance control. 

The intervention program using VR-based therapy consisted of 12 sessions of 45 minutes each, with two sessions a week. During the intervention period, the child underwent normal physical therapy sessions. The child attended all sessions. At each therapy session, the child had contact with two distinct games for a period of 20 minutes each, with a resting interval of five minutes between them. The two games used for the protocol were: a) A game in which the child saw himself projected inside an aquarium, which contained holes that had to be stopped using the upper or lower limbs; and b) A game in which the child, on top of a moving trailer, had to overcome obstacles by jumping, squatting and performing side-to-side movements of the body. 

## Results

Regarding baseline measures 1 and 2 (B1 and B2), the score values ​​in the tools used for the assessment remained the same. At the end of the intervention protocol, at baseline 3 (B3), an increase in the PBS score was observed, reaching the maximum score, as well as an increase in the MDS score, by increasing the general motor quotient (with the child going from a much inferior motor development to an inferior motor development). The areas of motor performance which showed an increase were: fine motor skills, overall motor function, balance, body schema and temporal organization. The data on the performance in each of the three assessments are shown in Table 1. 

**Table 1:**
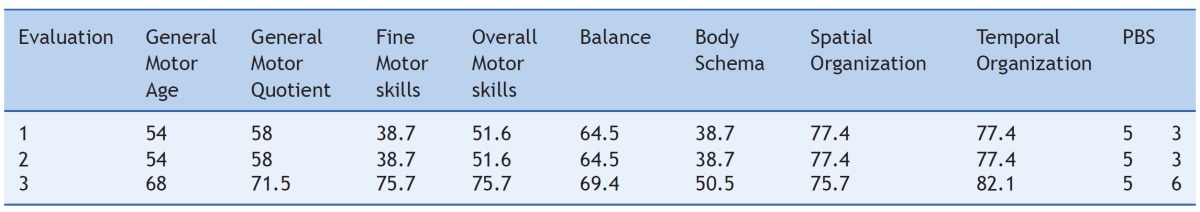
Score of the child MNS in each of the evaluations carried out using the MDS (Motor Development Scale) and PBS (Pediatric Balance Scale) tools

## Discussion

It was observed that the intervention protocol using VR resulted in gains on motor performance and functional balance in a child with CP and mild motor impairment. The increase in motor performance, verified through the MDS scale, promoted gains in all areas assessed by the tool, except spatial organization. This assessment also showed increased scores on the PBS tool, which after the intervention, reached the maximum score. 

Authors have verified improvement in balance and mobility in children with CP after VR-based therapies^18^^,^ 21 in tools such as the Community Balance and Mobility Scale^21^ and the Posture Scale Analyzer.^18^ Brien and Sveistrup^21^ used an intensive protocol of five consecutive days of contact with the VR-based therapy, noting improvements in balance after its use. However, VR is an adjuvant therapy,^11^ and therefore must have its effects evaluated on a weekly session program, which can be included in the routine treatment of patients and do not interfere with conventional therapy sessions. 

The present intervention used as a therapeutic resource a commercially available body scanning system, XBOX^(r)^360 Kinect^(r)^ (Microsoft(r), New York, United States). The motion sensor in the system can detect a comprehensive and accurate amount of movement in three dimensions and create a virtual image of the patient, which is projected on a screen.^12^ This projection provides real-time visual feedback of the performed movements,^13^ which allows the patient to see a representation of his movement pattern projected on the screen in real-time and correct compensatory movement patterns. This visual feedback, associated with verbal commands by the therapist, may have facilitated the adoption of postures with greater biomechanical alignment while performing the tasks. Thus, the constant repetition of tasks performed with continuous postural correction may have improved the child's postural pattern during games' playing, resulting in improved functional balance and gross motor performance. 

Studies using functional near-infrared spectroscopy (fNIRS) found that contact with the virtual environment provided by VR has the ability to increase blood perfusion in areas such as the superior temporal gyrus,^28^ which is responsible for balance, and activation in the primary motor cortex,^29^ which is responsible for motor performance. Thus, by increasing the activation of specific brain areas responsible for movement control, VR-based therapy produces cortical neuroplastic changes that are reflected in the child's motor gain in their daily activities.^29^

You *et al*,^29^ after four weeks of intensive therapy using VR in a hemiparetic child, verified cortical reorganization, with increased activation of bilateral brain areas responsible for performing motor gestures, such as the primary motor cortex and the sensory motor cortex. 

The capacity to activate brain motor areas important for movement control,^28^ associated with the child's immersion in the virtual environment through visual feedback, may have contributed to cortical reorganization after therapy. This potential reorganization may have determined the gains observed in global movement, balance, body schema and temporal organization in this case. Immersion in the virtual environment, combined with visual feedback, gave the child a better exploration of his body positioning in space, which resulted in the observed motor gains in his body schema. 

We also observed gains in the child's fine motor skills, possibly resulting from the consecutive repetition of movements of the upper limbs to meet the goals of the games selected for the therapy. One of the games required from the patient motor coordination and activation of the extensor and supinator muscles of the wrist to properly position his hand to stop the leaky points on the aquarium wall. The activation of these muscle groups can be especially difficult for children with hemiplegia, who have a flexion-pronation pattern of the upper limbs. Thus, the constant repetition of the movement, associated with the visual feedback of the video game and the therapist's verbal commands, enabled the patient to analyze and correct movement errors during the games, which may have reflected in his fine motor coordination. 

The repetition of motor gestures throughout the sessions may have promoted the transition from poorly coordinated actions and high cognitive demand to more effective actions, with the use of more functional patterns of movement (wrist in a neutral position without flexion of the fingers). According to the motor control theory, practice and feedback are important components for motor gains.^9^ Thus, the continuous repetition of motor gestures during the games throughout the intervention protocol therapy may have been responsible for the construction and coordination of new muscle synergies^21^ that influenced motor performance at the time of reassessment (B3). Furthermore, the demands of the tasks offered by the game required enhancement of temporal organization, through the need to perform motor gestures in time to meet the tasks proposed by the games and to be successful in them. 

The results showed no gains in spatial organization with the application of the VR-based protocol. Conceptually, spatial organization involves knowledge of the body dimensions and the space surrounding the body, including the ability to accurately assess the relationship between body and environment.^26^ The videogame works with components of the space-time notion;. however, at the end of the intervention, the child showed lower scores in this domain. Therefore, it can be considered that the contact with the videogame and the virtual environment was not sufficient to modify the schema of spatial organization assessed by the scale used in the study. The tasks evaluated in this domain of the scale involved some cognitive aspects, such as a test of fitting shapes into a board, comparing sticks of different sizes, and creating a rectangle with two triangles, tasks which are not specifically addressed by the games and require longer assimilation time than that of the intervention.^26^ It is also worth remembering that, compared to the gains obtained ​​in other dimensions, the score reduction was small and could be justified by a variation in the performance, which is not yet fully refined in a child aged seven. 

In this case, the choice of games used in the protocol and the specific demands of each may have contributed to the results. The games chosen for the intervention required the performance of diagonal movements of arms and legs, skill training to achieve specific targets, large range of motion movements, requiring the use of the trunk to generate weight transfers, squats and jumps. This interaction with the games chosen for the intervention challenged the child's motor repertoire, favoring his improvement both in relation to his overall balance and motor skills, as well as to his fine motor skills. 

The main limitations are related to the fact that this is a study of only one patient, with an intervention duration of only 12 sessions. Nevertheless, it can be concluded that the use of the VR-based therapy with a body scanning device resulted in positive effects on motor performance and functional balance of the analyzed child, who has CP with mild functional impairment. Thus, VR seems to be a promising tool to be incorporated into the rehabilitation process of CP. However, controlled studies in a group of patients and the assessment of the intervention efficacy, associated with image tests such as fNIRS are necessary, so that we can better understand the effects of VR-based therapy, especially on cortical organization. 
