# Supplementary material for: Impact of a virtual reality-based intervention on motor performance and balance of a child with cerebral palsy: a case study
Source: Rev Paul Pediatr. 2014 Dec;32(4):389–94. doi: 10.1016/j.rpped.2014.04.005 (PMC4311794; doi:10.1016/j.rpped.2014.04.005)
Supplement: Translation

# Translation: Impacto de intervenção baseada em realidade virtual sobre o desempenho motor e equilíbrio de uma criança com paralisia cerebral: estudo de caso

**Authors:** Silvia Leticia Pavão, Joice Luiza Bruno Arnoni, Alyne Kalyane Câmara de Oliveira, Nelci Adriana Cicuto Ferreira Rocha

**Affiliations:** Universidade Federal de São Carlos (UFSCar), São Carlos, SP, Brasil

**Keywords:** Paralisia cerebral, Criança, Equilíbrio postural, Desempenho psicomotor, Reabilitação, Terapia de exposição à realidade virtual

## Abstract

**OBJETIVO:**

O estudo buscou verificar o efeito de um protocolo terapêutico baseado em realidade virtual (RV) sobre o desempenho motor e o equilíbrio funcional de uma criança com paralisia cerebral (PC).

**DESCRIÇÃO DO CASO:**

Uma criança com PC hemiplégica espástica de 7 anos, nível de GMFCS I, foi submetida a um protocolo de intervenção fisioterapêutica de 12 sessões de 45 minutos, numa frequência de duas vezes semanais, com o uso de terapia baseada em RV. Utilizou-se um console comercialmente disponível (*XBOX(r)360 *Kinect(r)) capaz de rastrear a movimentação corporal, reproduzindo-a em uma tela. Anteriormente à intervenção, foram realizadas avaliações de seu desenvolvimento motor e equilíbrio por meio dos instrumentos: Escala de desenvolvimento Motor (EDM) e *Pediatric Balance Scale* (PBS), respectivamente. Foram realizadas duas avaliações com espaço de uma semana como linha de base e uma reavaliação após as 12 sessões. Não foram observadas diferenças nos escores dos instrumentos nas duas avaliações iniciais. Depois do protocolo de intervenção, o paciente aumentou o escore do instrumento PBS em três pontos, atingindo o teto da escala e, no instrumento EDM, passou de um desempenho motor muito inferior para apenas inferior.

**COMENTÁRIOS:**

As evidências apresentadas pelo presente relato apoiam o uso da RV como uma promissora ferramenta a ser incorporada no processo de reabilitação de paciente com disfunções neuromotoras.

## Introdução

As lesões encefálicas adquiridas, como as lesões hipóxico-isquêmicas até os três anos de idade estão entre as dez principais causas da paralisia cerebral (PC) hemiplégica espástica.^1^^-^ 2 O comprometimento motor do tipo hemiplegia, embora não comprometa de forma grave a funcionalidade da criança, produz alterações neuromotoras que acarretam déficits de precisão na realização de movimentos e déficits de controle postural, responsável pela estabilidade e o alinhamento entre os segmentos corporais durante a execução de atividades.^3^ Desta forma, a reabilitação de crianças com comprometimento motor leve, do tipo hemiplégico, pode mostrar-se especialmente desafiador aos terapeutas, exigindo conhecimento técnico profundo e criatividade.^4^^-^ 5

A presença de comprometimentos neuromotores na PC do tipo hemiplégica e sua elevada incidência na população infantil^5^ justificam o desenvolvimento de estudos que avaliem o efeito de intervenções terapêuticas sobre o equilíbrio e qualidade das respostas posturais destas crianças,^6^^-^ 8 com o objetivo de determinar as abordagens mais efetivas em sua recuperação funcional. Neste sentido, a terapia baseada na realidade virtual (RV) é uma ferramenta interativa de imersão dos pacientes em ambiente virtual que tem recebido destaque.^9^^-^ 11

A RV proporciona ao paciente *feedback *simplificado sobre a posição de seu corpo no espaço^12^ e lhe permite interatuar com os componentes virtuais em tempo real,^13^ estimulando a aprendizagem de estratégias de controle motor adaptativo em resposta a estímulos.^8^^,^ 14 A motivação e possibilidade de personalização da terapia proporcionada pelo contato com o ambiente virtual^15^^,^ 16 tornam-na uma importante ferramenta de reabilitação, que oferece experiências sensoriomotoras inviáveis em terapias comuns.

Estudos evidenciam melhora na função manual e na organização cortical em crianças com PC decorrentes de terapias baseadas na RV.^17^^-^ 18 Entretanto, embora existam estudos utilizando a terapia baseada na RV para crianças com PC, a maioria utiliza interfaces com o ambiente virtual na por meio de luvas^17^ ou controles com acelerômetros.^18^^-^ 20 Poucos estudos utilizaram a RV na reabilitação de crianças com PC por meio do uso de equipamentos comerciais de rastreamento da movimentação corporal.^21^^-^ 23

Equipamentos comerciais de rastreamento corporal, como o XBOX^(r)^360 Kinect, contêm sensores que captam a movimentação da criança em três dimensões, criando imagens que podem ser visualizadas pelo indivíduo na tela do televisor. Esta projeção permite a interação com o ambiente virtual por meio da movimentação corporal. Um recurso interessante, em vista do comprometimento de membros superiores presentes em crianças com PC, que poderiam inviabilizar o uso de luvas e controles remotos em seu tratamento. Chang *et al* 22 verificaram o efeito da terapia baseada na RV com o uso do XBOX(r)360 Kinect sobre a função manual de crianças com PC. Os autores utilizaram movimentos específicos para membros superiores, verificando efeitos positivos dessa modalidade terapêutica em crianças com comprometimento motor moderado. Luna-Oliva *et al* 23 também encontraram efeitos positivos do uso do Kinect como coadjuvante terapêutico sobre a função motora de crianças com comprometimento motor leve e moderado. 

Entretanto, embora estes estudos tenham demonstrando os benefícios da utilização de videogames com rastreamento da movimentação corporal como adjuvante terapêutico,^22^^-^ 23 as evidências encontradas ainda são limitadas, especialmente de sua utilização em crianças de baixo comprometimento funcional. De acordo com Campos *et al*,^4^ quanto menor o comprometimento motor observado em crianças com PC, mais difícil a obtenção de ganhos terapêuticos no processo de reabilitação. Desta forma, a progressão da terapia nestas crianças muitas vezes fica comprometida pela dificuldade em encontrar tarefas que as motivem e ao mesmo tempo apresentem eficácia terapêutica. Em vista disso, relata-se este caso clínico, no qual se avaliou o efeito de uma intervenção utilizando RV por meio do console comercialmente disponível XBOX^(r)^360 Kinect sobre o desempenho motor e o equilíbrio funcional de uma criança com PC de comprometimento motor leve. Embora se trate de uma avaliação preliminar, acredita-se que a imersão proporcionada pelo contato com ambiente virtual em conjunto com a motivação decorrente do contato com o videogame permitam à criança com comprometimento motor leve e altos níveis de funcionalidade desempenhar tarefas próximas às realizadas em sua rotina diária, facilitando a transposição do aprendizado motor gerado em terapia e levando a ganhos funcionais mensuráveis, aumentando sua inserção social no ambiente que a cerca.^14^^,^ 24

## Descrição do caso

MNS, sete anos, do sexo masculino, apresenta PC hemiplégica espástica direita nível I de acordo com o Sistema de Classificação da Função Motora Grossa (GMFCS),^25^ decorrente de uma lesão hipóxico-isquêmica aos dois anos de idade. A criança participa de um programa de intervenção fisioterapêutica baseada no Conceito Neuroevolutivo *Bobath* duas vezes por semana há quatro anos. Sua principal queixa é a dificuldade de participar de brincadeiras com saltos e transposição de obstáculos na escola com seus amigos.

A intervençao proposta foi aprovada pelo Comitê de Ética em Pesquisas com Seres Humanos da instituição em que a pesquisa ocorreu (processo nº 190.903), se em conformidade com a resolução 196/96 do Conselho Nacional de Saúde. O responsável pela criança autorizou sua participação no estudo assinando o Termo de Consentimento Livre e Esclarecido.

O utilizado envolveu o uso de medidas repetidas múltiplas de um único sujeito de acordo com o modelo A-B-A.^21^^-^ 23 Um fisioterapeuta treinado, cego no estudo, realizou todas as avaliações durante as três fases do estudo: linha de base 1 (B1), duas semanas anteriormente ao estudo; linha de base 2 (B2), no dia imediatamente anterior ao período de intervenção; linha de base 3 (B3), no dia seguinte após o término da intervenção. Foi realizada a avaliação da performance motora da criança, por meio da Escala de Desenvolvimento Motor (EDM)^26^ e o equilíbrio funcional por meio do *Pediatric Balance Scale* (PBS).^27^

O instrumento EDM, avalia as seguintes áreas do desenvolvimento: motricidade fina (IM1), motricidade grossa (IM2), equilíbrio (IM3), esquema corporal (IM4), organização espacial (IM5), organização temporal (IM6) e lateralidade. Com exceção da lateralidade, as demais áreas são avaliadas por dez tarefas cada uma, distribuídas em grau progressivo de dificuldade, entre as idades de 2 e 11 anos. A criança deve iniciar as tarefas, a partir da idade imediatamente inferior à sua. Cada tarefa completada com sucesso determina um ponto na Idade Motora (IM) correspondente. A partir do cálculo das idades motoras em cada área, realiza-se o cálculo de seu Quociente Motor Geral (QMG). Os valores do QMG são quantificados e categorizados, permitindo classificar as habilidades analisadas em padrões: muito superior (130 ou mais), superior (120-129), normal alto (110-119), normal médio (90-109), normal baixo (80-89), inferior (70-79) e muito inferior (69 ou menos).

O instrumento PBS, adaptado da Escala de Equilíbrio de Berg, avalia o equilíbrio em crianças com disfunções neuromotoras.^27^ É composto por 14 atividades, pontuadas entre zero e quatro, com zero indicando assistência máxima ou total para realizar a atividade e, quatro, a realização da tarefa independente. O escore total da escala é a soma de todas as pontuações, podendo variar de zero a 56, com os maiores escores indicando melhores habilidades no controle do equilíbrio.

O programa de intervenção utilizando terapia baseada em RV foi composto por 12 sessões de 45 minutos cada uma, numa frequência de duas sessões semanais. Durante o período de intervenção, a criança permaneceu normalmente em atendimento fisioterapêutico. A criança compareceu a todas as sessões. A cada sessão de terapia a criança tinha contato com dois jogos distintos por um período de 20 minutos cada um e um intervalo de descanso de cinco minutos entre eles. Os dois jogos utilizados para o protocolo foram: a) Um jogo em que a criança via-se projetada no interior de um aquário, no qual surgem constantes furos que deverem ser tampados com o uso de seus membros superiores ou inferiores; b) Um jogo no qual a criança, em cima de um trailer em movimento, deveri transpor obstáculos por meio de saltos, agachamento e deslocamentos látero-laterais do corpo.

## Resultados

Em relação às medidas da linha de base 1 e 2 (B1 e B2), os valores dos escores nos instrumentos utilizados para avalição permaneceram os mesmos. Ao término do protocolo de intervenção, na linha de base 3 (B3) foi observado aumento no escore do instrumento PBS, atingindo pontuação máxima, e um aumento na pontuação do instrumento EDM, por meio do aumento do quociente motor geral (com a criança passando de um desenvolvimento motor muito inferior a um desenvolvimento motor inferior). As áreas da performance motora em que foram observados aumentos foram: motricidade fina, motricidade global, equilíbrio, esquema corporal e organização temporal. Os dados de seu desempenho em cada uma das três avaliações encontram-se na tabela 1.

**Tabela 1:** Pontuação da criança MNS em cada uma das avaliações realizadas, por meio dos instrumentos EDM (Escala de Desenvolvimento Motor) e PBS (Pediatric Balance Scale)

## Discussão

Verificou-se que o protocolo de intervenção utilizando RV promoveu ganhos sobre o desempenho motor e o equilíbrio funcional na criança com PC de comprometimento motor leve. O aumento no desempenho motor, verificado por meio da escala EDM, promoveu ganhos em todas áreas avaliadas pelo instrumento, exceto organização espacial. Esta avaliação também permitiu observar o aumento da pontuação da criança no escore do instrumento PBS que, após a intervenção, atingiu a pontuação máxima.

Autores verificaram a melhora do equilíbrio e da mobilidade em crianças com PC após terapias baseadas em RV^18^^,^ 21 em instrumentos como *Comunity Balnce and Mobility Scale* 21 e o *Posture Scale Analyzer.* 18 Brien e Sveistrup21 utilizaram um protocolo intensivo de cinco dias consecutivos de contato com a terapia baseada em RV, observando melhoras no equilíbrio após o mesmo. Entretanto, a RV é uma ferramenta adjuvante à terapia^11^ e, portanto, necessita ter seus efeitos avaliados em um programa de sessões semanais, viáveis de serem inseridas na rotina de tratamento dos pacientes e que não atrapalhem sua terapia convencional. 

A presente intervenção utilizou como recurso terapêutico um sistema comercialmente disponível de escaneamento corporal (*XBOX* *(r)* *360 *Kinect(r)). O sensor de movimento neste sistema é capaz de detectar uma quantia abrangente e precisa de movimentos em três dimensões e criar uma imagem virtual do paciente projetada em tela.^12^ Esta projeção fornece *feedback* visual em tempo real dos movimentos realizados,^13^ o que permite ao paciente ver a representação do seu padrão de movimento projetada na tela em tempo real e corrigir padrões compensatórios de movimento. Este *feedback *visual, associado aos comandos verbais do terapeuta, pode ter facilitado a adoção de posturas com maior alinhamento biomecânico durante a realização das tarefas. Assim, a constante repetição de tarefas executadas com correção postural continua pode ter aprimorado o padrão postural da criança durante a execução dos jogos, refletindo-se em aprimoramento de seu equilíbrio funcional e seu desempenho motor grosseiro.

Estudos utilizando *functional near-infrared spectroscopy* (fNIRS) verificaram que o contato com o ambiente virtual proporcionado pelas RV é capaz de aumentar a perfusão sanguínea em áreas, como giro temporal superior,^28^ responsável pelo equilíbrio, e a ativação em córtex motor primário,^29^ responsável pela performance motora. Assim, ao aumentar a ativação de áreas cerebrais específicas responsáveis pelo controle do movimento, a terapia baseada na RV produz alterações corticais neuroplásticas, que se refletem no ganho motor da criança em sua atividades diárias.^29^

You *et al*,^29^ após quatro semanas de terapia intensiva por RV numa criança hemiparética, verificaram uma reorganização cortical com maior ativação de áreas bilaterais do cérebro, responsáveis pela execução de gestos motores, como o córtex motor primário e o córtex sensório motor. 

A capacidade de ativação de áreas motoras cerebrais importantes ao controle do movimento,^28^ associada à imersão da criança no ambiente virtual por meio do *feedback* visual pode ter contribuído para uma reorganização cortical após a terapia. Esta potencial reorganização pode ter determinado os ganhos observados em movimentação global, equilíbrio, esquema corporal e organização temporal no presente caso. A imersão em ambiente virtual, associada ao *feedback* visual, proporcionaram à criança uma melhor exploração do posicionamento de seu corpo no espaço, o que repercutiu nos ganhos motores observados em seu esquema corporal. 

Foram também observados ganhos na motricidade fina da criança, possivelmente decorrentes da consecutiva repetição de movimentos de membros superiores para cumprir com os objetivos dos jogos selecionados para terapia. Um dos jogos exigia do paciente coordenação motora e ativação da musculatura extensora e supinadora de punho, de forma a posicionar adequadamente sua mão ao encontro dos pontos de vazamento do aquário. A ativação destes grupos musculares pode ser especialmente difícil para crianças com hemiplegia, que apresentam um padrão de membros superiores em prono-flexão. Desta forma, a constante repetição do movimento, associada ao *feedback *visual do *vídeo game* e aos comandos verbais do terapeuta, possibilitaram ao paciente analisar e corrigir os erros de movimento durante os jogos, que pode ter se refletido em sua coordenação motora fina. 

A repetição dos atos motores ao longo das sessões pode ter possibilitado a transição de ações mal coordenadas e de alta demanda cognitiva para ações mais efetivas, com o uso de padrões mais funcionais de movimento (punho em posição neutra, sem flexão dos dedos). De acordo com a teoria de controle motor, prática e *feedback* são componentes importantes para ganhos motores.^9^ Desta forma, a repetição contínua de gestos motores durante os jogos, ao longo das terapias do protocolo de intervenção, pode ter sido responsável pela construção e coordenação de novas sinergias musculares^21^ que influenciaram a performance motora no momento de reavaliação (B3). Além disso, as demandas de tarefas oferecidas pelo jogo exigiram aprimoramento da organização temporal, pela necessidade de execução dos gestos motores a tempo de cumprir as tarefas propostas pelos jogos e alcançar sucesso nas mesmas. 

Os resultados encontrados não demonstraram ganhos na organização espacial com a aplicação do protocolo baseado em RV. Conceitualmente, organização espacial envolve o conhecimento das dimensões corporais e do espaço circundante ao corpo, incluindo a habilidade de avaliar com precisão a relação entre corpo e ambiente.^26^ O videogame trabalha componentes de noção espaço-temporal. Entretanto, ao final da intervenção, a criança apresentou menor pontuação neste domínio. Desta forma, pode-se considerar que o contato com o videogame e o ambiente virtual não foi suficiente para modificar o esquema de organização espacial avaliado pela escala utilizada. As tarefas avaliadas nesse domínio da escalas envolvem alguns aspectos cognitivos, tais como: prova de encaixar formas no tabuleiro, comparar palitos de diferentes tamanhos, montar um retângulo com dois triângulos, tarefas não trabalhadas especificamente pelos jogos e que requerem tempo superior aos da intervenção para assimilação.^26^ Vale lembrar ainda que, comparado aos ganhos apresentados nas demais dimensões, a redução do escore foi pequena, podendo justificar-se por uma variação de desempenho ainda não totalmente refinada em uma criança de sete anos. 

No presente caso, a escolha dos jogos utilizados no protocolo e a demanda específica de cada um podem ter contribuído para os resultados encontrados. Os jogos escolhidos exigiam a realização da movimentação de membros superiores e inferiores na diagonal, treino de destreza para atingir alvos específicos, movimentos de grande amplitude de movimento, requerendo a utilização de tronco para gerar transferências de peso, agachamentos e saltos. Esta interação com os jogos escolhidos desafiou o repertório motor da criança, favorecendo seu aprimoramento tanto em relação ao seu equilíbrio e motricidade global, quanto à sua motricidade fina.

As principais limitações relacionam-se ao fato de se tratar de um estudo de apenas um paciente, bem como seu tempo de intervenção de apenas 12 sessões. De qualquer forma, pode-se concluir que o uso da terapia baseada em RV, com um dispositivo de escaneamento corporal, produziu efeitos positivos sobre a performance motora e o equilíbrio funcional da criança analisada, portadora de PC de comprometimento funcional leve. Assim, a RV parece ser uma promissora ferramenta a ser incorporada no processo de reabilitação da PC. Entretanto,são necessários estudos controlados em um grupo de pacientes e a avaliação da eficácia da intervenção associada a exames de imagens, como o fNIRS, para que se possa melhor compreender os efeitos da terapia baseada na RV, especialmente na organização cortical.
